# Determination of the Membrane Environment of CD59 in Living Cells

**DOI:** 10.3390/biom8020028

**Published:** 2018-05-17

**Authors:** Gergő Fülöp, Mario Brameshuber, Andreas M. Arnold, Gerhard J. Schütz, Eva Sevcsik

**Affiliations:** Institute of Applied Physics, TU Wien, Wiedner Hauptstrasse 8-10, Vienna 1040, Austria; fulop@iap.tuwien.ac.at (G.F.); brameshuber@iap.tuwien.ac.at (M.B.); arnold@iap.tuwien.ac.at (A.M.A.); schuetz@iap.tuwien.ac.at (G.J.S.)

**Keywords:** plasma membrane, CD59, lipid, diffusion, micropatterning, GPI-anchored protein, membrane rafts

## Abstract

The organization and dynamics of proteins and lipids in the plasma membrane, and their role in membrane functionality, have been subject of a long-lasting debate. Specifically, it is unclear to what extent membrane proteins are affected by their immediate lipid environment and vice versa. Studies on model membranes and plasma membrane vesicles indicated preferences of proteins for lipid phases characterized by different acyl chain order; however, whether such phases do indeed exist in live cells is still not known. Here, we refine a previously developed micropatterning approach combined with single molecule tracking to quantify the influence of the glycosylphosphatidylinositol-anchored (GPI-anchored) protein CD59 on its molecular environment directly in the live cell plasma membrane. We find that locally enriched and immobilized CD59 presents obstacles to the diffusion of fluorescently labeled lipids with a different phase-partitioning behavior independent of cell cholesterol levels and type of lipid. Our results give no evidence for either specific binding of the lipids to CD59 or the existence of nanoscopic ordered membrane regions associated with CD59.

## 1. Introduction

For more than 20 years the question of how lipids influence the interaction of membrane proteins has remained unanswered. A particularly popular model describing protein–lipid interactions is the concept of membrane rafts—cholesterol-dependent nanoscopic membrane domains of higher lipid chain order serving to compartmentalize proteins and, thus, cellular functions. To date, however, no study has yielded unambiguous evidence for their existence in living cells. One problem is that protein–lipid interactions are inherently difficult to study in a live cell environment. Thus, many conclusions on the organization of proteins and lipids have been drawn from studies on model membranes and plasma membrane vesicles, where the coexistence of lipid phases of different fluidity—termed liquid-ordered (Lo) and liquid-disordered (Ld)—have been observed [1]. The evidence obtained in living cells, however, is often indirect and sometimes contradictory. In fact, in many studies, local and temporal heterogeneities of proteins and lipids have been found to arise from mechanisms other than lipid-mediated phase separation (reviewed in [2]).

Glycosylphosphatidylinositol-anchored proteins (GPI-APs) are a class of proteins attached to the outer leaflet of the plasma membrane via an anchor of usually saturated lipids [3]. Findings on their organization within the membrane, cellular sorting, and function have been inextricably intertwined with the raft hypothesis: based on the fact that GPI-APs are frequently isolated in detergent-resistant membrane (DRM) fractions and partition into ordered domains in phase-separated membrane blebs, they have been considered archetypical “raft markers” [4].

In cells, GPI-APs have frequently been reported to form cholesterol-dependent homo- and hetero-associates, but the properties of these associates were to some extent inconsistent as to their lifetime [1,5,6,7], actin-dependence [8,9,10], concentration-dependence [1,7], and mobility [1,7,11]. A recent study did not find evidence for pronounced homo- or hetero-co-diffusion of GPI-APs in fluorescence cross-correlation spectroscopy (FCCS) measurements [12]. Crosslinking of GPI-APs has been shown to recruit other raft markers and induce intracellular calcium flux, but here, too, observations have been contradictory [13,14,15] and mechanisms other than lipid-mediated phase separation have been described [16,17].

Since the proposition of the raft hypothesis in 1997 [18], many findings relating to the organization and dynamics of GPI-APs on the membrane, their sorting to the plasma membrane, and also their functions have been seen mostly in the context of the physicochemical properties bestowed by the GPI anchor: their status as “raft markers” took center stage. Only recently, Suzuki et al. [7] found that ectodomain interactions contributed to transiently stabilize GPI-AP homodimers. Still, the GPI anchor was proposed to be essential for forming larger and signaling-competent homo- and hetero-associates. This study, and many others, has used CD59 as an archetypical GPI-anchored protein in the sense that it was consistently found in DRMs [19] and shown to partition into Lo phase regions of giant plasma membrane-derived vesicles (GPMVs) [20]. These physicochemical properties of the GPI anchor of CD59 have been suggested to be key for its function in complement regulation: membrane rafts could act as platforms for the association of signaling molecules to transmit the signal from CD59 in the outer leaflet of the plasma membrane to the cell interior [21]. Other studies, however, have found no connection between the phase-partitioning behavior of membrane-anchored proteins and their ability to induce intracellular signaling events upon clustering [16].

Recently, we have developed an experimental approach that combines protein micropatterning and single molecule tracking to specifically address the role of lipid-mediated phase separation in the organization of GPI-APs [21]. We immobilized GPI-anchored monomeric green fluorescent protein (mGFP-GPI) in the plasma membrane of living cells within micrometer-sized patterns and used single molecule tracking to determine the effect of mGFP-GPI enrichment on the local membrane properties. We found that immobilized mGFP-GPIs did not act collectively by nucleating the formation of ordered regions in the plasma membrane but merely posed steric obstacles to the diffusion of other membrane constituents, thereby challenging major tenets of the raft hypothesis.

Here, we refine our previously introduced approach and apply it to probe lipid interactions of the GPI-anchored protein CD59. We monitor the diffusion of fluorescently labeled lipid analogs with a different phase-partitioning behavior in micropatterned cells and find that CD59 influences their mobility in a similar fashion. Our data show no evidence for specific binding of either of the two lipids to CD59. Further, our results allow us to rule out the formation of either micro- or nanoscopic ordered membrane regions in CD59-enriched areas. We did observe, however, a strong impact of the protein ectodomains on the diffusion behavior of membrane constituents, shifting the focus from the lipid bilayer part of the plasma membrane to the membrane-proximal protein-rich regions.

## 2. Results

### 2.1. Micropatterning Analysis Platform

**Figure 1:**
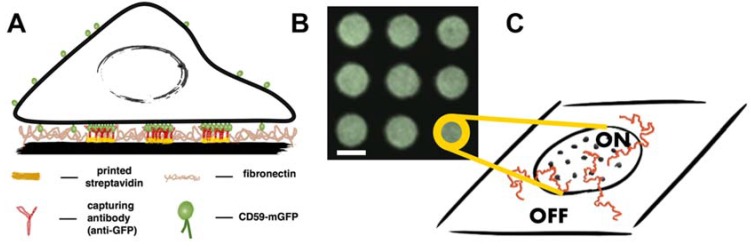
Principle of the experimental design and data analysis. (**A**) Antibody patterns are used to enrich and immobilize CD59-monomeric green fluorescent protein (mGFP) at specific sites in the plasma membrane of living T24 cells. Cells are interfaced with microstructured surfaces containing different densities of antibody, so that different surface densities of immobilized CD59-mGFP can be adjusted; (**B**) Typical region of interest in a micropatterned cell showing enrichment of CD59-mGFP in “ON” areas and depletion in “OFF” areas. Scale bar is 3 µm; (**C**) Sketch depicting individual immobilized CD59-mGFP molecules (dots) in “ON” areas. Trajectories of fluorescently labeled lipids (shown in red) are recorded and separated into the categories “ON” and “OFF” according to the CD59-mGFP patterns recorded in the GFP color channel.

We have recently developed a method for deliberate rearrangement of plasma membrane proteins using a micropatterning approach, where the surface of a glass coverslip is decorated with defined patterns of an antibody specific to an exoplasmic epitope of a membrane protein [21]. In the present study, patterns featuring an antibody against GFP were used to enrich and immobilize CD59-mGFP at specific sites in the plasma membrane of living T24 cells (Figure 1A). Cells interfaced with these structures showed a redistribution of CD59-mGFP within the plasma membrane to areas bearing antibody (“ON” areas), where it became immobilized. The density of immobilized CD59-mGFP, ρ_CD59-mGFP_, could be adjusted via application of different densities of GFP antibody and was determined for each cell by relating the pattern bulk brightness with the brightness of a single mGFP as described in [21]. The surface density of freely diffusing CD59-mGFP was the same in “ON” and “OFF” areas (Figure S1). Next, we incorporated different fluorescently labeled lipids into the plasma membrane of pattern-interfaced cells and recorded the diffusion of single molecules. We have previously shown that by comparing the localizations and mobilities of a diffusing tracer within protein-enriched areas (“ON”) and outside (“OFF”), conclusions on the interactions between tracer and obstacle can be drawn [21,22].

### 2.2. Diffusion of the Cholesterol Analog Chol-PEG-KK114 in Patterned Cells

Our main interest in this study was to determine whether CD59 influences its membrane environment with respect to nanoscopic membrane viscosity. One characteristic parameter of ordered membranes is the reduced mobility of its constituents compared to the disordered phase (~3-fold for GPMVs [23], ~10-fold for model membranes [24]). Bulky fluorophore labels at the lipid head group or in the acyl chain region usually prevent lipids from entering more tightly packed membrane areas such as the Lo phase of phase-separated model membranes [25,26]. Introduction of a flexible polyethylene glycol (PEG) linker has been shown to reconstitute much of the lipids’ phase partitioning behavior, without having a pronounced effect on their diffusion coefficients in model or cell membranes [27,28,29]. We therefore chose to use the cholesterol analog Chol-PEG-KK114, which slightly prefers the Lo phase (50% and 60% Lo partitioning in giant unilamellar vesicles (GUVs) and GPMVs, respectively [27]).

**Figure 2:**
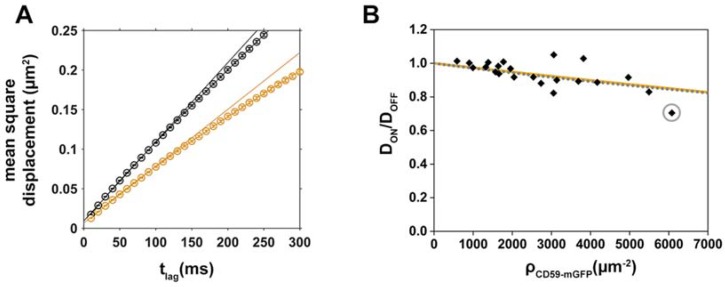
Mobility of the cholesterol analog Chol-PEG-KK114 decreases with increasing CD59-mGFP surface density. (**A**) Mean square displacements (msd) of “ON” (orange) and “OFF” (black) CD59-mGFPI areas are plotted as a function of t_lag_ for one representative cell. Diffusion coefficients were determined by fitting the function msd = 4Dt_lag_ + 4σ_xy_^2^; (**B**) Relative diffusion coefficients D_ON_/D_OFF_ were determined for individual cells (*N* = 22), plotted as a function of CD59-mGFP density, ρ_CD59-mGFP_, and fitted with Equation (2) (steric obstacles, orange line) or Equation (1) assuming a combined size of tracer and obstacle *d* = 2 nm (binding, dotted grey line). The gray circle indicates the cell shown in (**A**).

Single molecule trajectories of lipids, recorded in the red color channel, were separated into the categories “ON” CD59-mGFP pattern and “OFF” CD59-mGFP pattern by overlaying them with the CD59-mGFP pattern image, recorded in the GFP channel (Figure 1B). Figure 2A shows exemplary curves, where the mean square displacement (msd) of Chol-PEG-KK114 was plotted against the time lag (t_lag_), both for CD59-mGFP “ON” and “OFF” areas. Diffusion of Chol-PEG-KK114 could be well described by free Brownian motion in both areas. We determined the diffusion coefficients in “ON” and “OFF” areas and plotted the relative diffusion coefficients D_ON_/D_OFF_ as a function of the number density of CD59-mGFP, ρ_CD59-mGFP_, immobilized within patterns to create density/mobility plots (Figure 2B). We found that the relative mobility D_ON_/D_OFF_ of Chol-PEG-KK114 decreases with increasing CD59-mGFP density. Some D_ON_/D_OFF_ data points, particularly at low CD59-mGFP densities, exceed ratios of 1. This is the result of the variability of lipid diffusion constants within one cell, which depends on the location of the probed area. It is, however, independent from CD59-mGFP micropatterning: when we artificially divided Chol-PEG-KK114 trajectories recorded in nonpatterned cells into virtual “ON” and “OFF” fractions, we found D_ON_/D_OFF_ data ranging from 1.06 to 0.93 with a mean of 0.98 ± 0.06 (standard deviation, s.d.) (*N* = 21 cells). The diffusion coefficient of Chol-PEG-KK114 determined in “OFF” areas was 0.38 ± 0.03 µm^2^/s, a typical value for lipid diffusion in the plasma membrane of a living cell [27]. Table S1 shows a comparison of diffusion constants of the lipids used in this study in “OFF” areas of patterned cells and nonpatterned cells.

### 2.3. Analysis of CD59/Chol-PEG-KK114 Density/Mobility Data

Based on previous work by Saxton [30], we have recently performed in-depth Monte Carlo simulation studies on the effects that randomly distributed immobilized membrane proteins exert on the mobility of diffusing tracers [22]. In general, proteins act as steric obstacles, thereby reducing the mobility of tracer molecules. The obstacle density *C_P_*, at which long-range conducting paths for tracer diffusion disappear and the diffusion coefficient approaches zero, is termed the percolation threshold. Additional transient binding to the obstacles further reduces tracer mobility in a highly characteristic way, as described by

(1) $$\frac{D_{ON}}{D_{OFF}}=\left(1-\frac{1-e^{-\rho \cdot {\left(\frac{d}{2}\right)}^{2}\pi }}{C_{P}}\right)\left(1-\frac{1-e^{-\rho \cdot {\left(\frac{d}{2}\right)}^{2}\pi }}{{\bar{K}}_{D}+1-e^{-\rho \cdot {\left(\frac{d}{2}\right)}^{2}\pi }}\right)$$

with d, the combined size of obstacle and tracer; , the number density of immobilized obstacles; and $\bar{K_{D}}$, the dimensionless 2D binding constant $(\bar{K_{D}}$ = $K_{D}\pi {\left(\frac{d}{2}\right)}^{2})$ [22]. In our previous study [21], we based our calculations of obstacle and tracer sizes on a theoretical framework developed by Saxton [31], who calculated *C_P_* for various tracer sizes with respect to the obstacle size on a triangular lattice (regular matrix of hexagonal obstacles). Here, we use a model that does not rely on an a priori assumption of the relative sizes of tracer and obstacle to be able to determine their combined size. Randomly distributed circular obstacles with a diameter *d*_obs_ and tracers with *d*_tra_ can be simplified by assuming point tracers diffusing through a course of obstacles with a modified diameter *d* = *d*_obs_ + *d*_tra_ [30]. Modelling the obstacles as overlapping discs, the area coverage of obstacles at the percolation threshold is known and given by *C*_P_ ≈ 0.676 [32].

As a first approach, we assumed inert steric obstacles (K_D_→∞), which reduces Equation (1) to

(2) $$\frac{D_{ON}}{D_{OFF}}=\left(1-\frac{1-e^{-\rho {\left(\cdot \frac{d}{2}\right)}^{2}\pi }}{C_{P}}\right).$$

We determined the apparent diameter *d*_app_ of obstacle and tracer for the tracer/obstacle pair Chol-PEG-KK114/CD59-mGFP by fitting our experimental data with Equation (2), yielding 4.9 ± 0.5 nm (Figure 2B, orange curve). This size is larger than what would be expected for a diffusing cholesterol molecule sensing the lipid acyl chains of a GPI-AP as an obstacle (~2 nm).

It is possible that binding contributes to the observed decrease of mobility in CD59-mGFP areas. Thus, we assumed the smallest reasonable size for Chol-PEG-KK114/CD59-mGFP (2 nm) and fitted our data with a fixed *d* = 2 nm yielding a 2D dissociation constant K_D_ of ~40,000 molecules/μm^2^ (Figure 2B, dotted grey line). Our data fit equally well with a model assuming just steric obstacles and obstacles of a smaller size with a low binding affinity.

### 2.4. Disentangling the Components Affecting Lipid Diffusion on CD59-mGFP Patterns

**Figure 3:**
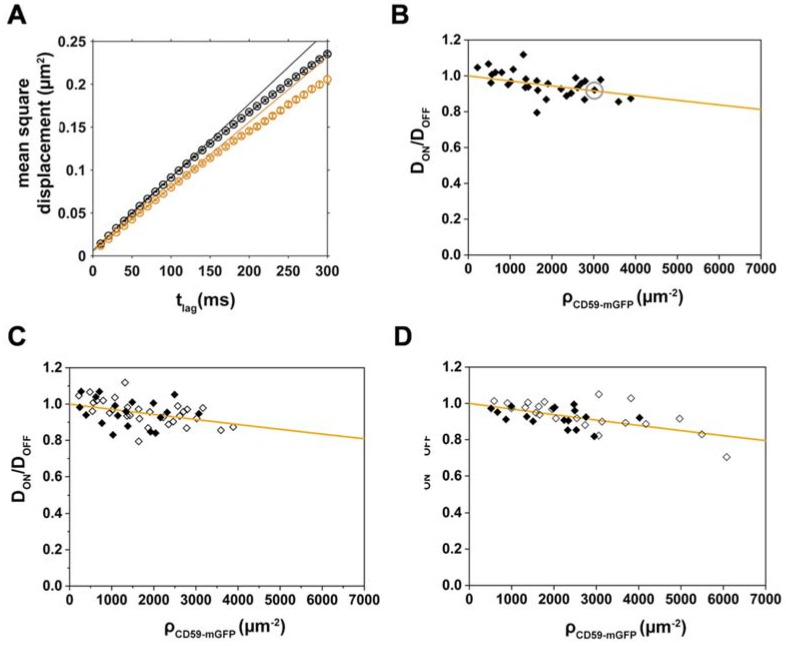
Dioleoyl-phosphatidylethanolamine(DOPE)-PEG-KK114 diffusion decreases with increasing CD59-mGFP density; the relative mobility of both lipids is independent of cell cholesterol levels. (**A**) Mean square displacements of DOPE-PEG-KK114 “ON” (orange) and “OFF” (black) CD59-mGFPI-enriched areas are plotted as a function of t_lag_ for one representative cell. Diffusion coefficients were determined by fitting the function msd = 4Dt_lag_ + 4σ_xy_^2^; (**B**) Relative diffusion coefficients D_ON_/D_OFF_ were determined for individual cells (*N* = 28), plotted as a function of CD59-mGFP density, ρ_CD59-mGFP_, and fitted with Equation (2) (orange line). The gray circle indicates the cell shown in (**A**). Relative diffusion coefficients D_ON_/D_OFF_ are plotted as a function of CD59-mGFP density (open diamonds) and after treatment with 1 U/mL cholesterol oxidase (closed diamonds) for (**C**) DOPE-PEG-KK114 (*N* = 19) and (**D**) Chol-PEG-KK114 (*N* = 17). Data from cholesterol-depleted cells were fitted with Equation (2) (orange line).

As described above, transient binding of Chol-PEG-KK114 to CD59-mGFP obstacles would cause an overall reduction of the diffusion in “ON” areas. By fitting Equation (2) in a case where binding was actually present, we would detect an apparently increased obstacle size *d*_app_. Thus, to determine whether specific interactions between Chol-PEG-KK114 and CD59-mGFP contribute to *d*_app_, we repeated our diffusion experiments using dioleoyl-phosphatidylethanolamine(DOPE)-PEG-KK114 as a tracer molecule (Figure 3A–C, Table S1). The apparent size we determined for the tracer/obstacle pair DOPE-PEG-KK114/CD59 was 5.0 ± 0.5 nm, similar to the value found for Chol-PEG-KK114. It is thus rather unlikely that specific binding between either lipid and CD59-mGFP contributes to tracer diffusion on CD59-mGFP patterns.

Another possible factor affecting Chol-PEG-KK114 diffusion on CD59-mGFP patterns would be the formation of a more viscous membrane environment in “ON” areas due to the high density of a “raft marker”. Dependence on acute cholesterol depletion has been taken as a hallmark for lipid-raft-associated processes. We thus used cholesterol oxidase to convert cholesterol to 4-cholesten-3-one, a sterol that inhibits Lo phase formation [33]. Cholesterol depletion, however, did not have a significant effect on the relative diffusion coefficients D_ON_/D_OFF_ of either DOPE-PEG-KK114 or Chol-PEG-KK114 (Figure 3C,D) yielding apparent sizes *d*_app_ of 5.0 ± 0.9 nm and 5.5 ± 0.6 nm, respectively.

## 3. Discussion

In this study, we found that CD59-mGFP influences the diffusion of fluorescently labeled analogs of cholesterol and DOPE in a similar fashion and determined apparent tracer/obstacle sizes of ~5 nm. This is larger than the size of the tracer/obstacle pair Chol-PEG-KK114/mGFP-GPI found in a previous study (2.4 nm), where d_app_ could be largely ascribed to steric hindrance in the acyl chain region [21]. There are several ways in which the mobility of lipids could be affected in CD59-mGFP-enriched “ON” areas: (i) CD59-mGFP acting as a steric obstacle to lipid diffusion; (ii) altered membrane viscosity as a result of a different nanoscopic membrane environment around the immobilized CD59-mGFP; and (iii) specific interactions, such as binding, between a lipid and CD59-mGFP. In the following we discuss the different possible contributions.

**Figure 4:**
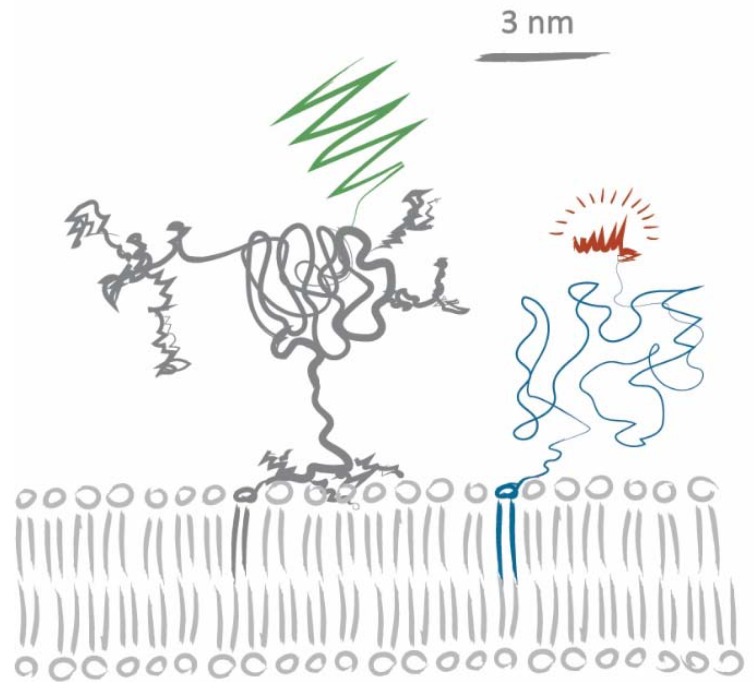
Sketch of CD59-mGFP and DOPE-PEG-KK114. We have used information from [27,34,35,37,38] for realistic size estimates of the CD59 core protein, its GPI anchor, and the C-terminally attached mGFP, as well as the lipid, the PEG linker, and the fluorophore.

Let us consider a scenario based on steric hindrance alone: human CD59 has an apparent mass of ~20 kDa, half of which arises from modifications of the core polypeptide through glycosylation and the GPI anchor linkage [34,35,36]. In the lipid analogs we used, the fluorophore is attached via a PEG linker to preserve phase partitioning properties of the lipids [29]. According to Flory’s theory, the average radii for PEG2000 (DOPE-PEG-KK114) and PEG3400 (Chol-PEG-KK114) can be estimated to be 2.8 nm and 3.8 nm, respectively [37]. Figure 4 shows a sketch of CD59-mGFP and DOPE-PEG-KK114 based on size information extracted from [27,34,35,37,38]. A tracer lipid only experiencing steric hindrance in the acyl chain region would yield a tracer/obstacle size of ~2 nm, while steric hindrance in the membrane-proximal ectodomain region would yield sizes of more than 10 nm (assuming rigid, cylindrical ectodomains). The apparent tracer/obstacle sizes we extracted from our data (~5 nm) are in between these two extremes. Certain aspects of our experimental approach and analysis could potentially influence our determined sizes: the modelling of CD59-mGFP as overlapping discs, incomplete chromophore maturation, and CD59 homodimerization. We have addressed each of these aspects in detail in Appendix A.

We also determined the size of the tracer/obstacle pair Chol-PEG-KK114/mGFP-GPI from [21] using Equation (2) yielding a d_app_ of 2.7 ± 0.5 nm, slightly larger than the 2.4 nm we estimated based on a triangular obstacle lattice and a priori assumptions on relative tracer/obstacle sizes. The difference in size of the Chol-PEG-KK114/mGFP-GPI and the Chol-PEG-KK114/CD59-mGFP pairs is most likely rooted in the larger size of the CD59 ectodomain and its extensive glycosylation. Interestingly, the glycosylation profile of the CD59 population is highly heterogeneous with more than 100 CD59 glycoforms within a single cell. The particular nature of its glycosylation has been proposed to influence the flexibility of CD59 relative to the GPI anchor [34], which may well affect the observed CD59-mGFP/tracer sizes. Other conceivable contributions to the determined sizes include interactions of the fluorophore or the PEG linker of the lipid tracers with the CD59 core protein, the GPI anchor, or the sugars. Also, we cannot rule out the possibility that CD59-mGFP is bound to (an) unknown protein(s), which is (are) co-enriched within “ON” pattern areas but invisible in our experiments and would thus generate an increased apparent tracer/obstacle size.

CD59 is an Lo marker and has been consistently isolated in DRMs [19,20]. This suggests that individual immobilized CD59-mGFPs may be surrounded by more viscous Lo nanodomains. The local enrichment of CD59-mGFP in “ON” areas could even induce the formation of a continuous more viscous membrane phase by recruiting lipids and proteins that favor such an environment. A locally higher membrane viscosity has different effects on different lipid tracers: First, a tracer that cannot enter such domains will experience an apparently increased obstacle size. Second, diffusion of a tracer that partitions into such nanodomains will be transiently slowed down. The two lipids we used in this study have different phase partitioning behavior: DOPE-PEG-KK114 prefers the liquid-disordered phase in GUVs and GPMVs (12% and 30% Lo partitioning, respectively) and Chol-PEG-KK114 slightly prefers the liquid-ordered (50% and 60% Lo partitioning, respectively) [27]. However, both lipids yielded very similar apparent tracer/obstacle sizes, allowing us to exclude the assembly of ordered-phase lipids and proteins within CD59-mGFP-enriched areas. Further, dependence on acute cholesterol depletion has been taken as a hallmark for lipid-raft-associated processes. Since neither the determined apparent size *d*_app_ of Chol-PEG-KK114 nor that of DOPE-PEG-KK114 was affected by depletion of cellular cholesterol, we can rule out an effect on *d*_app_ due to increased membrane viscosity in “ON” areas. Also, specific interactions are unlikely to play an important role because the determined apparent sizes were very similar for the two different lipids. Taking all this into account, our determined value of ~5 nm most likely reflects steric hindrance in the membrane-proximal region experienced by the diffusing lipids which is decreased by the flexibility of the PEG linker. The size limit for the detection of protein-associated membrane nanodomains with our approach is thus given by the steric hindrance of the ectodomains; we cannot rule out the existence of CD59-mGFP-associated nanodomains of a size of ~5 nm or smaller.

GPI-APs such as CD59 are usually considered in the context of the physicochemical properties of the GPI anchor. High local enrichment of CD59 produced by crosslinking with antibodies has been shown to induce cholesterol-dependent recruitment of other DRM-associated proteins and lipids which has been taken as an indication that the formation of CD59 clusters depended on raft-based lipid interactions [39,40]. We did not find any indication for the formation of more ordered membrane domains in areas of high CD59-mGFP density in our micropatterning experiments. Although not as tightly associated as in crosslinking experiments, CD59-mGFP covers 15% of the “ON” pattern area at a density of 5000 CD59-mGFP/μm^2^. One fundamental difference between most other studies and ours may be related to membrane curvature: GPI-APs are characterized by a bulky ectodomain anchored by a comparatively tiny lipid to the outer leaflet of the plasma membrane; it is conceivable that crosslinking of GPI-APs induces local membrane bending to form nanoscale buds as, e.g., observed for Cholera toxin-mediated crosslinking of the glycolipid GM_1_ [41]. Our micropatterning approach eliminates the aspect of membrane curvature as a variable from the experimental setup: by anchoring of CD59 to a glass coverslip, the plasma membrane is prevented from forming highly curved structures within CD59-mGFP patterns.

## 4. Materials and Methods

### 4.1. Micropattern Production

Microstructured surfaces were produced following a protocol described in [42]. Briefly, stamps featuring circular pillars with a diameter of 3 µm, center-to-center distance of 3 µm, and height of 2 µm were cleaned with absolute ethanol and dH_2_O, then incubated with 50 µg/mL streptavidin (AppliChem) in phosphate-buffered saline (PBS) (Sigma-Aldrich) for 15 min, rinsed with dH_2_O, and dried in a N_2_ flow. After drying, the stamp was placed on an epoxy-coated coverslip (Schott, Mainz, Germany) and incubated for 30 min at room temperature. After removal of the stamp, Secure-Seal hybridization chambers (Grace Biolabs, Bend, OR, USA) were placed on top of the structure and 10 µg/mL fibronectin (Sigma-Aldrich, Saint Louis, MO, USA) in PBS with 1% bovine serum albumin (BSA) (Sigma-Aldrich) was pipetted into the chamber. After 30 min incubation, the structures were rinsed with PBS and incubated with biotinylated GFP antibody (Novus, Littleton, CO, USA) at a concentration of 10 µg/mL in PBS with 1% BSA. After the antibody incubation, samples were rinsed thoroughly with PBS.

### 4.2. Cell Culture

The human bladder carcinoma cell line T24 (DSMZ no. ACC 310, Leibniz Institute DSMZ, Berlin, Germany) was stably transfected with CD59-mGFP and cultured in Roswell park memorial institute (RPMI) 1640 medium (Sigma-Aldrich) containing 10% fetal calf serum (FCS, Sigma-Aldrich). Cells were grown in a humidified atmosphere at 37 °C and 5% CO_2_. For micropatterning experiments, cells were grown to 60–80% confluency and harvested by treatment with accutase (Sigma-Aldrich). Cells were seeded onto the micropatterned surfaces and incubated for 45 min to ensure cell attachment before measurements. For cholesterol depletion, patterned cells were incubated in the presence of 1 U/mL cholesterol oxidase in Hank’s balanced salt solution (HBSS) at 37 °C for 20 min.

### 4.3. Total Internal Reflection Fluorescence Microscopy

Total internal reflection fluorescence microscopy experiments were performed on a home-built system including a modified inverted microscope (Zeiss Axiovert 200, Oberkochen, Germany) equipped with a 100 × oil-immersion objective (Zeiss Apochromat NA1.46, Oberkochen, Germany). For excitation of the Cholesterol-PEG-KK114 (Chol-PEG-KK114) and Dioleoyl-phosphatidylethanolamine-PEG-KK114 (DOPE-PEG-KK114), a 640 nm diode laser (Coherent, Obis, Santa Clara, CA, USA) was used. For excitation of CD59-mGFP, a 488 nm diode laser (Toptica, ibeam-smart, Munich, Germany) was applied. Emission light was filtered using appropriate filter sets (Chroma, Bellow Falls, VT, USA) and recorded on an iXon DU 897-DV EM-CCD camera (Andor, Belfast, Ireland). Total internal reflection fluorescence (TIRF) illumination was achieved by shifting the excitation beam in parallel to the optical axis with a mirror mounted on a motorized movable table.

### 4.4. Determination of CD59-mGFP Density

The surface density of CD59 immobilized in “ON” areas was essentially determined as described in [21]. All measurements with cells were performed in HBSS solution supplemented with 2% FCS at 23 °C. The CD59-mGFP density, *ρ*, specifies the number of CD59-mGFP molecules per µm^2^; it was determined by relating the mean bulk brightness of the CD59-mGFP -enriched (ON) areas with the brightness of single CD59-mGFP molecules. In order to determine the single molecule brightness, we performed a variant of the thinning out clusters while conserving stoichiometry of labeling (TOCCSL) method developed in our lab [1] on CD59-mGFP-expressing cells seeded on a fibronectin-coated coverslip. Briefly, a rectangular region of interest was completely photobleached by a strong laser pulse. After a recovery time of 5 s, we recorded sequences of 100 images, which contained well-separated signals of single CD59-mGFP molecules. For determination of the single molecule brightness we discarded the first 10 images to avoid detection of CD59-mGFP dimers. In several of these image sequences we identified single molecule signals and used in-house-developed Matlab (MathWorks, Natick, MA, USA) scripts based on a maximum likelihood estimator to determine position, integrated brightness, full width at half-maximum (FWHM), and local background of individual signals [1]. The median of the intensity values was taken for the single mGFP brightness.

We determined the number of immobilized CD59-mGFP proteins for every region, where single molecule diffusion was measured. The mean pixel intensity values of “OFF” area regions were subtracted from the mean pixel intensity values of “ON” regions to determine the intensity contribution of immobilized CD59-mGFP species. The resulting mean pixel intensity of immobilized CD59-mGFP was divided by the single molecule brightness to yield the surface density *ρ*_mGFP-CD59_. We verified that binding by antibodies did not change GFP brightness: the mean brightness of nonpatterned T24 CD59-mGFP cells (*N* = 9) before and 15 min after incubation with biotinylated GFP antibody under saturating conditions (20 µg/mL) was 4798 ± 2171 (s.d.) and 4581 ± 2027 (s.d.) counts per pixel, respectively.

The surface density of freely diffusing CD59-mGFP was determined by measuring the recovery of CD59-mGFP fluorescence 5 minutes after bleaching a defined area of a micropatterned cell. The mean brightness of “ON” and “OFF” areas within the photobleached area was determined for 15 spots on 5 different cells.

### 4.5. Single Molecule Tracking and Diffusion Analysis

Micropatterned cells were incubated with either Chol-PEG(3400)-KK114 (20 ng/mL, 10 min) or DOPE-PEG(2000)-KK114 (20 ng/mL, 10 min) in HBSS containing 2% FCS and washed thoroughly after the incubation. First, a CD59-mGFP pattern image was acquired in the GFP channel, then a series of movies were recorded at an illumination time of 3 ms and a frame rate of 10 ms in the red channel. Images were analyzed using MATLAB algorithms modified from [43]. To determine probe diffusion coefficients respective to the CD59-mGFP pattern, trajectories were divided into the two fractions “ON” and “OFF” using selection masks made from the underlying CD59-mGFP images. The mean square displacements (msd) were plotted against the time lag and the diffusion coefficient was determined by fitting the function msd = 4Dt_lag_ + 4σ_xy_^2^, where σ_xy_ denotes the localization precision; diffusion coefficients were determined from the first two data points [44]. Msd vs t_lag_ plots showed a minor deviation from pure Brownian motion at higher time lags. This is because with increasing time lag there is an increasing fraction of tracers leaving “ON” and “OFF” areas defined by the selection mask, which cannot be analyzed and hence do not contribute to the results. The consequence is a bias towards slower-diffusing tracers. We have verified this by applying a typical selection mask to tracer diffusion data recorded on nonpatterned cells (Figure S3). The diffusion coefficients determined from the first two data points in the msd vs t_lag_ plot remained unaffected by application of the selection mask and hence reliably report on the correct diffusion behavior. From the probe diffusion coefficients—D_ON_ and D_OFF_—we calculated the relative diffusion coefficient D_ON_/D_OFF_ and plotted it against CD59-mGFP surface density *ρ*_CD59- mGFP_. Data were fitted in Origin using Equation (1) or Equation (2) as indicated in the main text.

### 4.6. Flow Cytometry

T24 wild-type and CD59-mGFP-positive cells were detached from the culture plates by treating them with accutase (Sigma-Aldrich). Cells were then washed with precooled staining buffer (HBSS containing 2% FCS) and incubated on ice for 30 min with 30 μg/mL anti-CD59-Alexa Fluor™ 647 (MEM-43) (AbD Serotec, Hercules, CA, USA), then washed twice with the staining buffer stored on ice prior to experiments. Cells were used at a concentration of 6 × 10^5^ cells/staining. Samples were analyzed on an SH800S Cell Sorter (Sony, Tokyo, Japan) and the data were further processed with the FlowJo software (Treestar, Ashland, OR, USA). Living single cells were gated according to their forward- and side-scatter characteristics.

## Supplementary Materials

The following are available online at http://www.mdpi.com/2218-273X/8/2/28/s1. Figure S1: Determination of the surface density of freely diffusing CD59-mGFP. Figure S2: Estimation of the relative expression levels of endogenous CD59 in T24 CD59-mGFP cells. Figure S3: Effect of application of a selection mask on msd vs t_lag_. Table S1: Apparent lipid/CD59-mGFP sizes d_app_. Table S2: Diffusion of lipids in patterned and nonpatterned cells.

## Appendix A

Further considerations on the determined apparent sizes.

**Applicability of the model.** Membrane proteins and lipids may be more realistically modelled as soft disks interacting by a power-law repulsive potential than overlapping discs [30]. For soft disks, the determination of *C_P_* is not straightforward and has a weak dependence on the combined size of obstacle and tracer. The *C_P_* value for soft disks with *d* ≈ 5.5 nm was determined to be 0.7539 [30]. Using this *C_P_* value, the sizes of Chol-PEG-KK114/CD59-mGFP and DOPE-PEG-KK114/CD59-mGFP changed only in a minor way to 5.2 ± 0.4 nm and 5.3 ± 0.5 nm, respectively (Table S2).

**Chromophore maturation.** One factor that also needs to be considered when estimating obstacle sizes is incomplete chromophore maturation of mGFP, which can result in an underestimation of the obstacle density. Considering incomplete maturation of up to 20% [45] we determine slightly smaller sizes with *d*_app_ for Chol-PEG-KK114/CD59-mGFP and DOPE-PEG-KK114/CD59-mGFP being 4.4 ± 0.4 nm and 4.5 ± 0.4 nm, respectively (Table S2).

**Transient homodimers.** CD59 has been reported to form transient cholesterol-dependent homodimers [7]. If this happens between an immobilized CD59-mGFP and a mobile CD59-mGFP, this will not markedly affect our data as both are detected as individual obstacles. T24 cells constitutively express CD59, which will not be immobilized within patterns by the GFP antibody and may be transiently trapped at CD59-mGFP obstacles, thereby increasing its apparent size. Indeed, dark, endogenous CD59 could amount to as much as 1/5 of the total CD59 in our cells (Figure S1). It is unlikely, though, that transient formation of cholesterol-dependent homodimers is contributing to the apparent obstacle size since the apparent tracer/obstacle sizes we determined in untreated and cholesterol-depleted cells were similar.
