# Supplementary material for: Determination of the Membrane Environment of CD59 in Living Cells
Source: Biomolecules. 2018 May 17;8(2):28. doi: 10.3390/biom8020028 (PMC6023084; doi:10.3390/biom8020028)
Supplement: Supplementary file 1 [file biomolecules-08-00028-s001.pdf]

biomolecules

MDPI

Supplementary Information

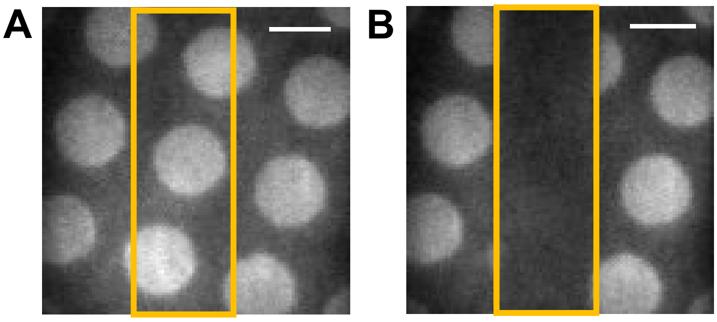

Figure S1. Determination of the surface density of freely diffusing CD59-mGFP. Fluorescence images of a ROI in a representative patterned T24 CD59-mGFP cell before **(A)** and 5 minutes after **(B)** bleaching an area defined by a field stop (indicated by the yellow rectangle). The mean brightness of "ON" and "OFF" areas after photobleaching was 5625 ± 267 (s.d.) and 5575 ± 163 (s.d.), indicating that the surface density of freely diffusing CD59-mGFP recovering into the bleached area is essentially the same in "ON" and "OFF" areas. Scale bars are 3 µm.

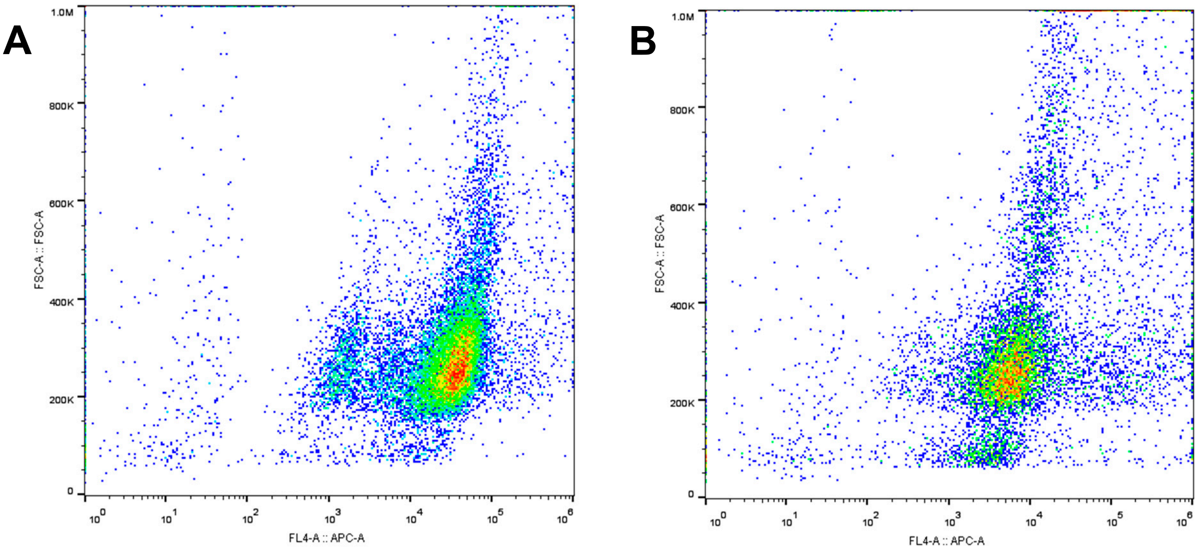

Figure S2. Estimation of the relative expression levels of endogenous CD59 in T24 CD59-mGFP cells. **(A)** Flow cytometry data of T24 CD59-mGFP and **(B)** T24 wildtype cells labeled with anti-CD59-Alexa Fluor™647. The fluorescence intensity in the red color channel is plotted vs. the forward scattering. The mean fluorescence intensities per cell were 3.7×10^4 for the T24 CD59-mGFP cells and 6.6×10^3 for T24 wildtype cells. Based on these values we estimate the fraction of "dark" endogenous CD59 in CD59-mGFP-positive cells to be lower than 1/5 of total CD59.

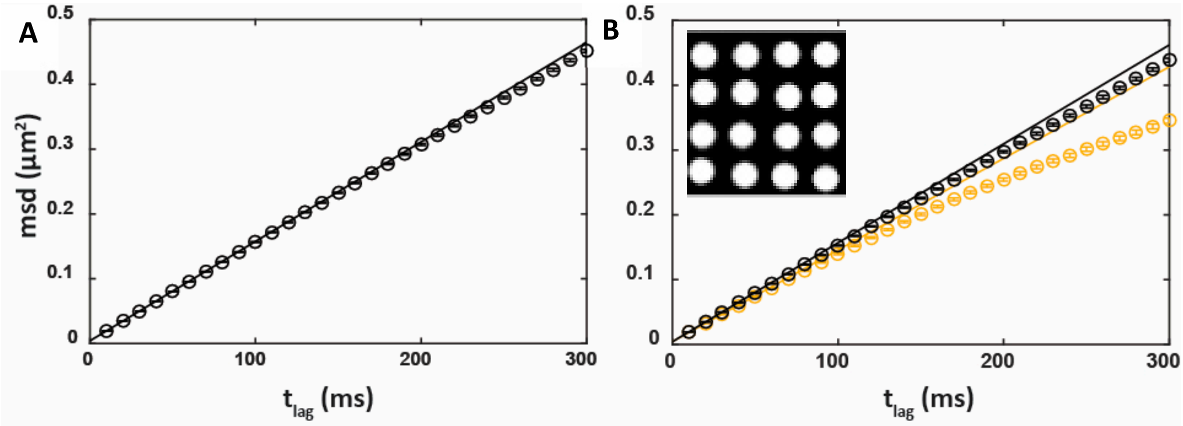

Figure S3. Effect of application of a selection mask on msd vs. t_{lag} plots. Msd vs. t_{lag} plots of Chol-PEG-KK114 diffusion recorded in a non-patterned cell without **(A)** and with **(B)** application of a typical selection mask generated from CD59-mGFP patterns to separate "ON" (yellow) and "OFF" (black) trajectories. The selection mask is shown as an insert in **(B)**; white areas in the mask correspond to "ON" areas, black to "OFF" areas. The diffusion coefficients determined from the first two data points without selection mask, in "ON" and in "OFF" areas were 0.38 ± 0.01 µm²/s, 0.37 ± 0.01 µm²/s and 0.38 ± 0.01 µm²/s, respectively.

Table S1. Diffusion of lipids in patterned and non-patterned cells. Diffusion of lipids was recorded and determined in "OFF" areas in patterned cells and unpatterned cells. Standard errors of the mean are indicated.

	**Chol–PEG-KK114**	**DOPE–PEG-KK114**
Unpatterned cells	0.343 ± 0.036[1]	0.405 ± 0.036
"OFF" areas	0.379 ± 0.028	0.411 ± 0.032

[1] Diffusion constant in µm²/s.

Table S2. Apparent lipid/CD59-mGFP sizes d_{app}. Apparent sizes of tracer/obstacle pairs were determined by fitting data from Figures 2B and 3B-D with Eq.2. Standard errors (SE) are indicated; the 95% confidence interval is given by d_{app} ± (1.96×SE).

	**untreated cells**	**chol depleted**	**untreated cells soft disc model**	**incomplete chromophore maturation**
Chol-PEG-KK114	4.9 ± 0.4[1]	5.5 ± 0.6	5.2 ± 0.4	4.4 ± 0.4
DOPE-PEG-KK114	5.0 ± 0.5	5.0 ± 0.9	5.3 ± 0.5	4.5 ± 0.4

[1] d_{app} is given in nm.
